# Challenging Molecular Diagnosis of Congenital Adrenal Hyperplasia (CAH) Due to 21-Hydroxylase Deficiency: Case Series and Novel Variants of *CYP21A2* Gene

**DOI:** 10.3390/cimb46050291

**Published:** 2024-05-16

**Authors:** Paola Concolino

**Affiliations:** Dipartimento di Scienze di Laboratorio ed Ematologiche, UOC Chimica, Biochimica e Biologia Molecolare Clinica, Fondazione Policlinico Universitario Agostino Gemelli IRCCS, Largo A. Gemelli 8, 00168 Rome, Italy; paola.concolino@policlinicogemelli.it; Tel.: +39-0630154250; Fax: +39-0630156706

**Keywords:** congenital adrenal hyperplasia (CAH), 21-hydroxylase deficiency (21OHD), molecular diagnosis, *CYP21A2*, RCCX CNV

## Abstract

Congenital adrenal hyperplasia (CAH) is a group of autosomal recessive genetic defects in cortisol synthesis and shows elevated ACTH concentrations, which in turn has downstream effects. The most common variant of CAH, 21-hydroxylase deficiency (21OHD), is the result of pathogenic variants in the *CYP21A2* gene and is one of the most common monogenic disorders. However, the genetics of 21OHD is complex and challenging. The *CYP21A2* gene is located in the RCCX copy number variation (CNV), a complex, multiallelic, and tandem CNV in the major histocompatibility complex (MHC) class III region on chromosome 6 (band 6p21.3). Here, *CYP21A2* and its pseudogene *CYP21A1P* are located 30 kb apart and share a high nucleotide homology of approximately 98% and 96% in exons and introns, respectively. This high-sequence homology facilitates large structural rearrangements, copy number changes, and gene conversion through intergenic recombination. There is a good genotype–phenotype correlation in 21OHD, and genotyping can be performed to confirm the clinical diagnosis, predict long-term outcomes, and determine genetic counseling. Thus, genotyping in CAH is clinically relevant but the interpretations can be challenging for non-initiated clinicians. Here, there are some concrete examples of how molecular diagnosis can sometimes require the use of multiple molecular strategies.

## 1. Introduction

More than 95% of cases of congenital adrenal hyperplasia (CAH), an autosomal recessive condition, are caused by pathogenic variants affecting the gene encoding the 21-hydroxylase enzyme (P450c21), which catalyzes the conversion of 17-hydroxyprogesterone and progesterone to 11-deoxycortisol and deoxycorticosterone, respectively [1,2]. The 21-hydroxylase deficiency (21OHD) (OMIM # 201910) is characterized by poor cortisol production, elevated ACTH levels, and the subsequent accumulation of precursor steroid hormones in the steroidogenic pathway, resulting in hyperandrogenism [1,2]. The disorder is categorized into two main clinical phenotypes: the severe or classical and the mild late-onset or non-classical (NC). The classical phenotype comprises two early-onset forms reflecting the degree of aldosterone deficiency: the salt-wasting (SW) form, which represents the most severe and potentially life-threatening expression of the disease, and the simple virilizing (SV) form [3,4].

The severe classic form of 21OHD affects 1 of 15,000 and 1 of 200–1000 in its mild NC form, with asymptomatic carrier prevalence estimated as 1/60 and 1/11, respectively [5,6,7,8].

In the human leukocyte antigen (HLA) class III region, on the short arm of chromosome 6p21.3, four tandemly arranged genes—serine/threonine kinase *RP*, complement *C4*, steroid 21-hydroxylase *CYP21*, and tenascin *TNX*—form a genetic unit designated as an RCCX segment [9]. In an RCCX haplotype with two segments, duplication of the RCCX occurs and the orientation of genes, from telomere to centromere, is *RP1-C4A-CYP21A1P-TNXA-RP2-C4B-CYP21A2-TNXB* [9].

The *CYP21A2* gene, exclusively expressed in the adrenal cortex, encodes the steroid 21-hydroxylase enzyme, whereas the *CYP21A1P* pseudogene encodes a non-functional protein inactivated by small insertions/deletions and pathogenic single-nucleotide variants. Both the *CYP21A2* and the *CYP21A1P* consist of a total of ten exons spanning 3.4 kb with a sequence identity of 98% and around 96% in their exons and intronic regions, respectively [10,11].

Approximately 75% of the *CYP21A2* pathogenic variants are transferred by conversion (microconversion when the event involves only one variant or large conversion when more pseudogene variants are involved) from the pseudogene during meiosis, while only 5–10% of pathogenic alleles harbor *CYP21A2* variants that do not result in gene conversions [12,13,14,15,16]. In contrast, 20–25% of the cases of 21OHD are related to large misalignments due to unequal crossing over during meiosis. These events may cause gene deletion or amplification, and also broader deletions involving the *CYP21A2* gene and the other contiguous genes [9,14,15,16,17]. For example, the *CYP21A1P/CYP21A2* chimeric gene is the result of a recombination between *CYP21A1P* and *CYP21A2* genes as an unequal crossing over occurs during meiosis [18]. Copy number changes in the RCCX segment are also the effect of unequal crossover. The most well-known case is a haplotypic RCCX CNV structure containing three distinct segments with two *CYP21A2* gene copies and one *CYP21A1P* pseudogene copy [19,20]. Finally, an unequal crossing over between *TNXA* and *TNXB* genes produces a chromosome with two copies of the *CYP21A2* gene and a chromosome where the arrangement of the RCCX segment shows the *C4-CYP21A1P-TNXA/TNXB* sequence, lacking the *CYP21A2* gene copy. This condition is associated with CAH-X Syndrome [21,22].

There are many studies reporting the frequency of the most common *CYP21A2* pathogenic variants in different populations [23]. In addition, a good genotype–phenotype correlation has been recognized, even if there is well-documented evidence that divergence can occur in some cases. In this regard, the classic example is represented by the c. 293-13A/C>G pathogenic variant [24].

The complexity of the RCCX region, the short sequence transfers, and the large structural rearrangements complicate the molecular diagnosis of 21OHD. Here, three peculiar cases were selected. These were challenging to extricate and required the simultaneous use of multiple molecular strategies in order to identify the pathogenic variants and study their segregation within families. Despite these difficulties, molecular diagnoses were successfully performed, and all genotypes identified were concordant with the severe phenotype exhibited by the probands. In two specific cases, novel *CYP21A2* pathogenetic variants were identified.

## 2. Methods

All clinical investigations and genetic analyses were performed according to the guidelines of the Declaration of Helsinki. Molecular analyses were commissioned to the Molecular Diagnostic Unit of Policlinico A. Gemelli by external hospitals where the patients were diagnosed with CAH. Written informed consent was obtained from all subjects.

### 2.1. Copy Number Variation (CNV) Detection

Multiplex ligation-dependent probe amplification (MLPA) assay was employed to establish the exact copy number of the *CYP21A2* gene (SALSA MLPA Kit P050-D1 CAH; MRS Holland, Holland, the Netherlands). The SALSA MLPA Probemix P050-D1 CAH contains 30 MLPA probes with amplification products between 130 and 382 nucleotides. This includes eight probes for the *CYP21A2* gene and four probes for the *CYP21A1P* pseudogene. The *CYP21A2* probes detect the wild-type sequences of seven loci: the c.-113G>A variant in the promoter region, the c. 293-13A/C>G within intron 2 (two wild-type alleles, C and A, at this location, for both of which a probe is present), the c.332_339del (p.Gly111fs) in exon 3, the c.518T>A (p.Ile173Asn) in exon 4, the c.713T>A (p.Val238Glu) and the c.719T>A (p.Met240Lys) in exon 6, and the c.923dup (p.Leu308fs) in exon 7.

A minimum of three reference samples were included in each experiment. Coffalyser.Net software (v.240129.1959) was used for data analysis. The following cut-off values for the final ratio (FR) of the probes were used to interpret the MLPA result: 0 copies (FR = 0), 1 copy (0.40 < FR < 0.65), 2 copies (0.80 < FR < 1.20), and 3 copies (1.30 < FR < 1.65).

To facilitate the interpretation of the results obtained by MLPA, refer to Table 1, which illustrates the *CYP21A2* and *CYP21A1P* probes available in the SALSA MLPA Kit P050-D1 CAH.

### 2.2. Analysis of the CYP21A2 Downstream of the TNXB Gene

CYP779f (5′-ccagaaagctgactctggatg-3′; located in the 5′ end of the *CYP21A1P* and *CYP21A2* genes) and Tena32F (5′-ctgtgcctggctatagcaagc-3′; located in a nonduplicated area of *TNXB* in exon 32) primers were used to amplify an 8.5-Kb PCR fragment, containing the 5′-end of the *CYP21A2* and partial *TNXB* genes, according to a protocol by Lee et al. [25]. This PCR product was directly sequenced (internal primers available on request) using the BigDye Terminator Cycle Sequencing Kit, Version 3.1 (Thermo Fisher Scientific, Waltham, MA, USA) and an ABI 3500 Genetic Analyser (Thermo Fisher Scientific, Waltham, MA, USA) according to the manufacturer’s instructions. Sequencing electropherograms were analyzed against the reference sequence NM_000500.9 using the SeqScape Version 4.0 software package (Thermo Fisher Scientific, Waltham, MA, USA).

### 2.3. Detection of the CYP21A2 Downstream of the TNXA Gene

This protocol was previously reported [20,26]. Briefly, a 6.1 Kb PCR product containing whole *CYP21A1P* and partial *TNXA* genes was amplified using CYP-779f (5′-aggtgggctgttttcctttca-3′) and XA36 F (5′-ggacccaga aactccaggtgg-3′) primers. Successively, 500 ng of this PCR product was used for enzymatic restriction with the TaqI enzyme and, after incubation at 65 °C for 2 h, the completely digested PCR products were analyzed by electrophoresis on a 1.0% agarose gel. When a *CYP21A2* gene was present downstream of the *TNXA* gene, the restriction pattern of the 6.1-Kb PCR amplicon showed fragments of 3738, 3207, 2315, 591, and 60 bp. Thus, the 3738 bp fragment, including the whole sequence of the *CYP21A2* gene downstream of the *TNXA* gene, was subsequently isolated from agarose gel (QIAEX II Gel Extraction Kit, Qiagen, Hilden, Germany) and sequenced using internal primers. A second strategy to isolate the *CYP21A2* gene downstream of the *TNXA* was to perform two nested PCR on 6.1-Kb fragment using specific, previously described, *CYP21A2* primers: CYP5 (5′-agctataagtggcacctcagg-3′; located in the 5′ end of the *CYP21A1P* and *CYP21A2* genes)/PROMR (5′-agcagggagtagtctcccaag-3′) (fragment 1) and P3 (5′-ttgtccttgggagactactcc-3′)/XA36F (5′-ggacccaga aactccaggtgg-3′) (fragment 2). P3 and PROMR primers are specific to the *CYP21A2* gene since they target the wild-type sequence of exon 3 where an 8 bp deletion (c.332_339del; p.Gly111fs) maps in the pseudogene [20]. Sequencing of both fragments was performed using internal primers and the results were analyzed using the SeqScape Version 4.0 software package (Thermo Fisher Scientific, Waltham, MA, USA).

## 3. Case Series

### 3.1. Case 1

**Figure 1:**
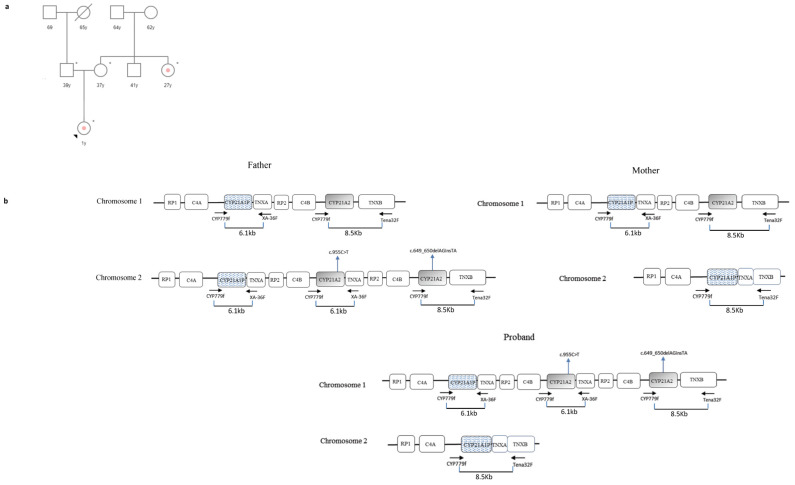
Case 1. (**a**) Genetic pedigree of family 1. The affected individuals are marked with a dot. The arrow points to the proband. y: years; * tested subjects; (**b**) *CYP21A2* haplotypes of the proband and her parents. The father carried a chromosome with three RCCX segments where the *CYP21A2* next to *TNXB* carried the novel c.649_650delAGinsTA (p.Arg217Ter) variant and the *CYP21A2*-like gene downstream of the *TNXA* harbored the c.955C>T (p.Gln319Ter) mutation. The mother carried a chromosome with a *CYP21A2* deleted allele and a chimeric *TNXA/TNXB* gene. The proband inherited the deleted chromosome from the mother and the chromosome with the novel haplotype from the father. No experiments were performed to establish the exact structure of the RCCX haplotypes (presence or absence of HERV-K(C4) insertion and type of *C4* gene in each segment). In the figure, *C4* may be *C4A* or *C4B*. *RP1*: Serine/Threonine Kinase 19; *RP2*: Serine/Threonine Kinase 19 pseudogene; *C4*: Complement component 4; *CYP21A1P*: Steroid 21-Hydroxylase Pseudogene; *TNXA*: Tenascin XA Pseudogene; *CYP21A2*: Steroid 21-Hydroxylase; *TNXB*: Tenascin XB. Variants are numbered in relation to the *CYP21A2* reference sequence NM_000500.9.

The patient was a 1-year-old girl diagnosed with SW CAH a few days after birth due to an adrenal crisis and ambiguous genitalia presentation. She was the first daughter of non-consanguineous parents of Peruvian origins resident in central Italy (Figure 1a). No history of CAH was reported in the paternal family, while a maternal aunt, a 27-year-old woman, was diagnosed with NC CAH at age eight (Figure 1a). Blood samples from the proband, both parents, and the maternal aunt were available for genetic analysis.

In order to establish the exact copy number of the *CYP21A2* gene, MLPA was performed as the first level of the analysis on all subjects. Successively, as reported in the methods section, specific PCR-based amplification strategies were used for variant detection.

Father: MLPA analysis detected three copies of the *CYP21A2* gene and two copies of the *CYP21A1P* pseudogene. In fact, the restriction pattern of the 6.1-kb fragment (obtained using CYP779f and XA-36F primers) revealed the presence of a duplicated *CYP21A2* allele downstream of the *TNXA* gene (Figure 1b).

The amplification of the 8.5-Kb fragment (using CYP779f and Tena32F primers) was used as a strategy for characterizing the *CYP21A2* gene next to the *TNXB* and the sequencing identified a heterozygous variant in exon five, the c.649_650delAGinsTA (p.Arg217Ter) (Figure 1b). At the same time, in order to isolate the duplicate *CYP21A2* allele downstream of the *TNXA* gene, two nested PCRs (using CYP5/PROMR and P3/XA36F primers) were performed on the 6.1-Kb fragment, and the successive sequencing of these two products revealed the c.955C>T; p.(Gln319Ter) variant in exon eight (Figure 1b).

The presence of a *CYP21A2* wild-type allele (located next to the *TNXB*) made the man unaffected; however, this first analysis did not establish whether the two *CYP21A2* alleles carrying the pathogenic variants were located on the same chromosome or they lay on different chromosomes. Only the subsequent segregation analysis clarified this aspect (Figure 1b).

Mother: the MLPA assay detected two copies of *CYP21A1P* pseudogene and only one *CYP21A2* allele. In this case, the 8.5-Kb PCR (CYP779f and Tena32F primers) amplified both a fragment containing the pseudogene and part of the chimeric *TNXA/TNXB* gene (on the deleted chromosome) and the only *CYP21A2* allele on the other chromosome (Figure 1b). For this reason, it was necessary to isolate the *CYP21A2* allele contained in the 8.5 kb PCR fragment by two nested PCRs: two genomic DNA fragments were amplified using two sets of specific primers: CYP5 (5′-agctataagtggcacctcagg-3′)/P2R (5′-gcatctccacgatgtga-3′) (fragment1) and P3 (5′-ttgtccttgggagactactcc-3′)/P4 (5′-acctctcgcacccccagtatgact-3′) (fragment 2). P2R and P3 primers are specific to the *CYP21A2* gene since they target the wild-type sequence of exon 6 (where a cluster of three missense pathogenic variants, Exon6 Cluster, p.Ile237Asn, p.Val238Glu, and p.Met240Lys, is present in the pseudogene) and exon 3 (where the 8 bp deletion is present in the pseudogene), respectively. The sequencing of these two fragments did not reveal the presence of pathogenic variants, confirming that the woman carried a wild-type *CYP21A2* allele (Figure 1b). In fact, according to her genotype, she was unaffected.

Proband: MLPA analysis detected two copies of the *CYP21A2* gene and two copies of the *CYP21A1P* pseudogene. However, the sequencing of the 8.5-Kb PCR (CYP779f and Tena32F primers) identified heterozygous pseudogene variants, raising suspicions that the child had inherited the deleted chromosome from her mother (Figure 1b). The isolation of the *CYP21A2* allele from the 8.5-Kb product (using the same strategy performed on the mother) and the successive sequencing revealed the c.649_650delAGinsTA (p.Arg217Ter) paternal variant (Figure 1b). At this point, if the proband carried a deleted maternal chromosome and the MLPA detected two copies of the active gene, the second *CYP21A2* allele must have been on the paternal chromosome next to the *TNXA* gene. In fact, the nested PCR, performed on the 6.1-Kb fragment (using the same strategy performed on the father), allowed us to isolate this duplicated *CYP21A2* allele and discover the c.955C>T; p.(Gln319Ter) variant (Figure 1b). The proband inherited a deleted chromosome from her mother and a chromosome with two copies of the *CYP21A2* gene, both mutated, from her father (Figure 1b). This genotype explains the severe phenotype.

Maternal aunt: the woman was hemizygous for the p.Val282Leu variant. In fact, MLPA analysis detected only one copy of the *CYP21A2* gene. This genotype was compatible with her NC CAH phenotype.

### 3.2. Case 2

A female child of non-consanguineous Italian parents was born at 39 weeks of gestation with normal delivery and an APGAR score of 10. On initial examination, the neonate was assigned as male based on the phenotypic appearance of the external genitalia. However, after the first 12 days of life, the newborn was diagnosed with the SW CAH and reassigned as female based on biochemical findings and karyotype results. The neonate was the third child born to this family, and no other close relative was reported with similar clinical issues.

**Figure 2:**
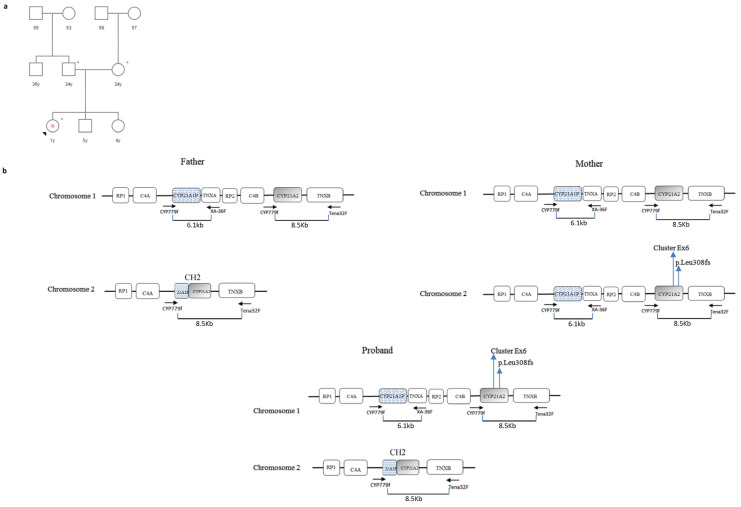
Case 2. (**a**) Genetic pedigree of family 2. The affected individuals are marked with a dot. The arrow points to the proband. y: years; * tested subjects; (**b**) *CYP21A2* haplotypes of the proband and her parents. The father carried a chromosome with a chimeric *CYP21A1P/CYP21A2* gene (CH2 chimera) while the mother carried a *CYP21A2* allele harboring Exon6 Cluster mutations and the p.Leu308fs variant within exon 7. The proband inherited the chimeric gene from the father and the mutated allele from the mother. No experiments were performed to establish the exact structure of the RCCX haplotypes (presence or absence of HERV-K(C4) insertion and type of *C4* gene in each segment). In the figure, *C4* may be *C4A* or *C4B*. *RP1*: Serine/Threonine Kinase 19; *RP2*: Serine/Threonine Kinase 19 pseudogene; *C4*: Complement component 4; *CYP21A1P*: Steroid 21-Hydroxylase Pseudogene; *TNXA*: Tenascin XA Pseudogene; *CYP21A2*: Steroid 21-Hydroxylase; *TNXB*: Tenascin XB. Variants are numbered in relation to the *CYP21A2* reference sequence NM_000500.9.

**Figure 3:**
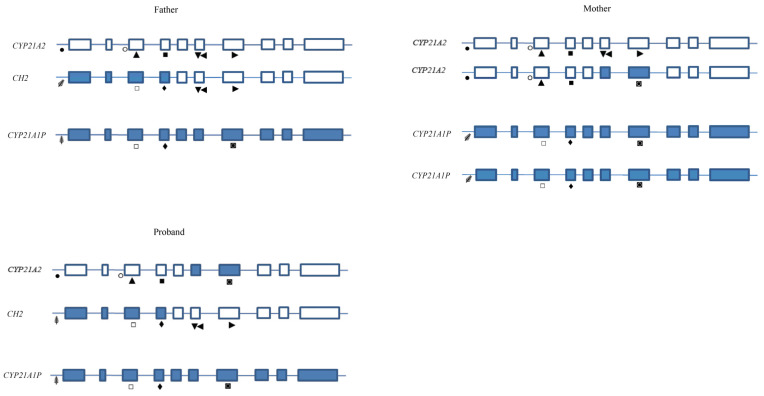
Family 2 genotypes. The father carried a wild-type *CYP21A2* gene, a chimeric *CYP21A1P/CYP21A2* gene (CH2), and a *CYP21A1P* allele. The mother carried a wild-type *CYP21A2* gene, a *CYP21A2* allele with Exon6 Cluster mutations (exon 6) and the p.Leu308fs variant (exon 7), and two copies of *CYP21A1P* gene. The proband inherited the chimeric *CYP21A1P/CYP21A2* gene (CH2), the mutated *CYP21A2* allele, and one copy of *CYP21A1P* pseudogene. The symbols under the exons symbolize annealing of the MLPA probes. To associate a specific probe with each symbol, refer to Table 1. Proband MLPA data interpretation: *CYP21A2* probes: ● This probe detected the wild-type sequence of the *CYP21A2* promoter region. The FR was <0.80 because a single copy was identified. ○ This probe detected the wild-type allele C of *CYP21A2* intron 2. The FR was >0.80. In fact, when a single copy of allele C is present, this probe shows a score between 0.80 and 1.2. ▲ This probe detected the wild-type sequence of the *CYP21A2* exon 3. The FR was <0.80 because a single copy was identified. ■ This probe detected the wild-type sequence of the *CYP21A2* exon 4. The FR was <0.80 because a single copy was identified. ▼ This probe detected the wild-type sequence of *CYP21A2* exon 6. The FR was <0.80 because a single copy was identified: CH2 exon 6. ◄ This probe detected the wild-type sequence of *CYP21A2* exon 6. The FR was <0.80 because a single copy was identified: CH2 exon 6. ► This probe detected the wild-type sequence of *CYP21A2* exon 7. The FR was <0.80 because a single copy was identified: CH2 exon 7. CYP21A1P probes: ⸙ This probe detected the pseudogene promoter region. The FR was >0.80 because two copies were identified: *CYP21A1P* gene e CH2 gene. □ This probe detected the pseudogene exon 3 region. The FR was >0.80 because two copies were identified: *CYP21A1P* gene e CH2 gene. ♦ This probe detected the pseudogene exon 4 region. The FR was >0.80 because two copies were identified: *CYP21A1P* gene e CH2 gene. ◙ This probe detected the pseudogene exon 7 region. The FR was >0.80 because two copies were identified: *CYP21A1P* gene e *CYP21A2* mutated gene.

Blood samples from the proband and both parents were available for genetic analysis. In order to facilitate understanding of the MLPA results, please refer to Figure 2 and Figure 3 and Table 1.

Father: The FR of *CYP21A2* 15221-L20261 (exon 3), 22959-L32396 (exon 4), and 22964-L32402 (promoter region) probes was <0.80 (normal range 0.80 < FR > 1.20), indicating a heterozygous deletion of these regions. Similarly, the *CYP21A1P* 17261-L21170 (exon 7) probe showed an FR of 0.53 (Figure 3, Table 1).

The sequencing results of the 8.5 kb PCR led to the suspicion of a chimeric gene or a large conversion event. In fact, the pseudogene sequence up to exon 4 of the gene was detected, identifying the following heterozygous variants: c.-126C>T, c.-113G>A, c.-110T>C, c.-103A>G (promoter region), p.(Pro31Leu) (exon 1), c.293-13A/C>G (intron 2), p.(Gly111fs) (exon 3), and the p.(Ile173Asn) (exon 4).

In order to isolate the wild-type *CYP21A2* allele, two sets of specific primers CYP5/PROMR (fragment1) and P3/P4 (fragment 2) were used to amplify genomic DNA. In fact, PROMR and P3 primers are specific to the *CYP21A2* gene by targeting the wild-type sequence of exon 3 (where the p.Gly111fs variant is present in the pseudogene). As expected from the patient’s phenotype, no mutations were detected during the sequencing. Combining the MLPA results with those of sequencing, it was deduced that the man carried a wild-type *CYP21A2* allele “in trans” with a *CYP21A1P/CYP21A2* chimeric gene (the previously reported CH2 chimera, [27]) (Figure 2b).

Mother: The FR of *CYP21A2* MLPA 17261-L21169 (exon 7), 17270-L16990 (exon 6), and 17271-L16989 (exon 6) probes was <0.80 (normal range 0.80 < FR > 1.20), indicating a heterozygous deletion of these exons. In addition, the *CYP21A1P* 17261-L21170 (exon 7) probe showed an FR value of 1.42 miming the duplication of exon 7 (Figure 3, Table 1). The sequencing of the 8.5-Kb PCR detected the following heterozygous variants: the Exon6 Cluster in exon 6 and the p.(Leu308fs) in exon 7 (Figure 2b). The presence of these variants prevented the annealing of *CYP21A2* MLPA probes, which recognize the wild-type sequences of the respective exons. For this reason, the FR was <0.80. Differently, the pseudogene exon 7 probe 17261-L21170, specific to the p.(Leu308fs) variant, also bound the exon 7 of the gene (harboring the p.Leu308*fs* mutation) showing an FR value of 1.42 (Figure 3, Table 1).

Since the woman was asymptomatic and her hormonal values were within the normal range, it was likely that the two variants were on the same allele, while a second wild-type allele was present on the other chromosome. For this reason, it was decided to proceed with the analysis of the proband and not to carry out further investigations on the mother.

Proband: While pseudogene probes were all within normal range, all *CYP21A2* MLPA probes showed an FR < 0.80 (Figure 3, Table 1). Without sequencing and segregation analysis data, this result could be interpreted as deletion of the whole *CYP21A2* gene. Instead, the 8.5 Kb PCR sequencing identified heterozygous pseudogene variants confirming the presence of two *CYP21A2* alleles (Figure 2b).

In order to isolate the chimeric paternal allele, two sets of specific primers were used: CYP5/P2R and P2F (5′-agggatcacatcgtggagat-3′)/P4, where P2R and P2F bind the wild-type sequence of *CYP21A2* exon 6 (where the Exon6 Cluster is present in the pseudogene). Differently, to isolate the maternal allele, PCR was performed using CYP5/PROMR and P3/P4-specific primers, where PROMR and P3 primers recognize the wild-type sequence of *CYP21A2* exon 3 (where the p.Gly111fs is present in the pseudogene). In this way, it was possible to establish that the child had inherited a chimeric allele (CH2) from her father and an allele with the Exon6 Cluster mutations and the p.Leu308fs variant from her mother (Figure 2b). This agreed with her severe phenotype.

### 3.3. Case 3

**Figure 4:**
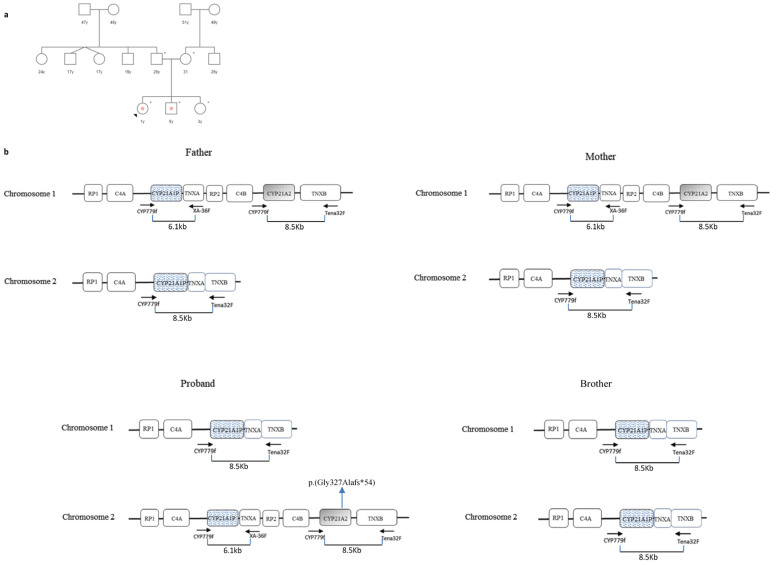
Case 3. (**a**) Genetic pedigree of family 3. The affected individuals are marked with a dot. The arrow points to the proband. y: years. * tested subjects. (**b**) *CYP21A2* haplotypes of the proband, her parents, and her brother. Both parents carried a chromosome with a deleted *CYP21A2* allele and a chimeric *TNXA/TNXB* gene. The brother inherited both deleted chromosomes from his parents while the proband carried the novel de novo c.980_981delGC (p.Gly327Alafs*54) variant in hemizygosis. No experiments were performed to establish the exact structure of the RCCX haplotypes (presence or absence of HERV-K(C4) insertion and type of *C4* gene in each segment). In the figure, *C4* may be *C4A* or *C4B*. *RP1*: Serine/Threonine Kinase 19; *RP2*: Serine/Threonine Kinase 19 pseudogene; *C4*: Complement component 4; *CYP21A1P*: Steroid 21-Hydroxylase Pseudogene; *TNXA*: Tenascin XA Pseudogene; *CYP21A2*: Steroid 21-Hydroxylase; *TNXB*: Tenascin XB. Variants are numbered in relation to the *CYP21A2* reference sequence NM_000500.9.

The proband was the third born of non-consanguineous parents of Indian origins residing in northern Italy (Figure 4a). She was born at gestational week 38 (birth weight, 2100 g, karyotype 46, XX) with ambiguous genitalia (Prader III), showing mild generalized hypotonia, poor head control, and difficulty in feeding. After hormonal evaluation, she was diagnosed with classical SW CAH and replacement steroid therapy (hydrocortisone and fludrocortisone) was administered. The couple’s first child, aged 5, was also diagnosed at birth with SW CAH, while the second born was healthy. Blood samples from the whole family were sent to our laboratory for genetic investigation.

MLPA analysis detected only one copy of the *CYP21A2* gene in both parents (all MLPA probes showed an FR < 0.50). No pathogenic variants were identified isolating this *CYP21A2* allele from the 8.5 kb PCR (Figure 4b). Furthermore, no copy of the *CYP21A2* gene was detected in the proband’s first brother, who inherited a deleted chromosome from each of his parents and exhibited a severe 21OHD phenotype (Figure 4b). Differently, the healthy sister carried two wild-type *CYP21A2* alleles.

In the proband, while the MLPA assay identified a single copy of the *CYP21A2* gene (all probes showed an FR < 0.50), the sequencing showed a novel de novo variant within exon 8, the c.980_981delGC frameshift mutation (Figure 4b).

## 4. Discussion

Nonallelic homologous recombination (NAHR) plays a key role in RCCX genetic diversity: large structural rearrangements, copy number changes, and gene conversion are the consequence of the peculiar co-presence of genes and pseudogenes with high sequence homology and are responsible for specific human diseases such as 21OHD [9].

The challenges related to the molecular diagnosis of 21OHD are well documented [28,29,30,31,32], and for this reason, molecular biologists refer to specific protocols for genetic testing investigating *CYP21A2* defects. Best-practice genotyping is PCR-based sequence analysis along with MLPA, which detects most types of potential alterations [33]. For this purpose, the first evidence consists of the accurate choice of a PCR strategy to perform an allele-specific amplification. As reported, most laboratories amplify one or more *CYP21A2* fragments, covering all 10 exons and the respective exon/intron boundaries, by selective PCR primers differentiating the functional *CYP21A2* gene from the *CYP21A1P* pseudogene followed by Sanger sequencing [33]. The most frequently used strategy consists of amplifying two overlapping fragments that cover the whole sequence of the *CYP21A2* gene. In the first fragment, the forward primer is nonspecific (it recognizes the promoter sequence of both the gene and the pseudogene) while the reverse primer recognizes a wild-type sequence of exon 6 where a cluster of three missense mutations is present in the pseudogene. Similarly, in the second fragment, while the reverse primer recognizes a 3′UTR region of both the gene and the pseudogene, the forward primer is specific to the gene, being built in a region of exon three of the gene where an 8-base deletion is instead present in the pseudogene [34]. However, this strategy not only creates a concrete risk of dropout (since mutations in exons three and six of the pseudogene may have been transferred to the gene through conversion and avoid primer binding), but it is not even able to discriminate between the different *CYP21A2* alleles that may be present in a haplotype with multiple RCXX segments. For example, in the case of duplicated *CYP21A2* alleles, the risk of no correct assignment of the variants cannot be excluded. In addition, even if two *CYP21A2* copies are detected by MLPA, this could be due to a duplication of the *CYP21A2* gene on one chromosome, whereas the second chromosome is lacking a functional *CYP21A2* allele, resulting in a CAH-carrier state. To avoid these mistakes, it is necessary to selectively amply the *CYP21A2* allele downstream of the *TNXB* gene by a long PCR protocol producing a fragment of 8.5 Kb, as reported in the method section of this report. Nevertheless, it is necessary to consider that, as a consequence of a 30 kb deletion, the structure of the RCCX region may change, and downstream of *TNXB,* there could lie a chimeric *CYP21A1P/CYP21A2* gene or, when the deletion affects the whole *CYP21A2* gene, a chimeric *TNXA/TNXB* gene. In these cases, the 8.5 kb PCR product contains these chimeric genes [9].

Similarly, a long PCR protocol giving a 6.1 Kb fragment allows us to isolate the *CYP21A1P* allele downstream of the *TNXA* gene (as reported in the method section). However, some RCCX haplotypes have additional structures where, downstream of the *TNXA* gene, there is an extra *CYP21A2* allele (*CYP21A2*-like gene), which is amplified by 6.1 kb PCR [20,35,36].

Due to the complexity of RCCX CNV, MLPA should be used as a first step in the analysis to determine the exact copy number of the *CYP21A2* gene and *CYP21A1P* pseudogene. However, as previously reported, the interpretation of MLPA data is particularly difficult and requires a profound understanding of 21OHD genetics [29]. In summary, we must be aware that the standardization of methods in the case of 21OHD is complicated and that all methods mentioned so far may have limitations and none of those techniques might be able to identify 100% of possible variants. For this reason, in order to resolve the most complicated cases, it is necessary to implement different strategies during the course of the molecular investigation.

Here, a series of peculiar cases have been successfully resolved allowing the identification of genetic defects. In family 1, segregation analysis revealed the complex genotype of the proband: MLPA analysis identified two copies of the *CYP21A2* gene; however, they were located on the same paternal chromosome while the maternal *CYP21A2* allele was deleted. The *CYP21A2* allele downstream of *TNXA* carried the c.955C>T (p.Gln319Ter) variant while the copy present next to the *TNXB* harbored the novel c.649_650delAGinsTA variant (Figure 1b). This variant is characterized by a two-nucleotide deletion of adenine (A) and guanine (G) at positions c.649 and c.650, respectively, followed by the insertion of thymine (T) and adenine (A) at the same locus. These substitutions create a premature stop codon (p.Arg217Ter) leading to a truncated non-functional protein. In fact, the c.649_650delAGinsTA variant was predicted as “likely pathogenic” by VarSome Software (v.240129.1959) [37].

In case 2, MLPA analysis detected only one copy of the *CYP21A2* gene in the proband. However, the child carried a chimeric *CYP21A1P/CYP21A2* gene on the paternal chromosome and one allele with the Exon6 Cluster and the p.Leu308fs variant on the maternal chromosome (Figure 2b). This is a typical example of the challenging interpretation of MLPA data and how these must always be considered in combination with the results obtained from the sequencing analysis.

Finally, case 3 represented a combination of unfortunate events that led the proband, the daughter of non-consanguineous parents, to inherit a chromosome with a *CYP21A2* deleted allele while a novel de novo mutation arose on the second allele (Figure 4b). The c.980_981delGC variant was predicted to cause a frameshift in the reading frame of the *CYP21A2* gene, leading to an altered amino acid sequence downstream of the deletion site. This frameshift resulted in a premature stop codon truncating the protein (p.Gly327Alafs*54) and potentially leading to a loss of normal enzyme function. This variant was also predicted as “likely pathogenic” by VarSome Software (v.240129.1959) [37].

## 5. Conclusions

21OHD represents one of the most intriguing topics in genetics. The challenge of 21OHD molecular diagnosis is related to the complexity of the RCCX CNV structure, a complex, multiallelic, and tandem CNV located in the major histocompatibility complex (MHC) class III region. Here, the genetic diversity is supported by nonallelic homologous recombination (NAHR): unequal crossover facilitates large structural rearrangements and copy number changes, whereas gene conversion mediates relatively short sequence transfers. The results of these events point out the molecular basis of 21OHD.

Currently, modern massively parallel sequencing techniques have revolutionized the field of molecular diagnostics. However, the molecular diagnosis of 21OHD requires the use of standard methods (Long PCR, Sanger sequencing, and MLPA), the development of different strategies, and deep expertise in the interpretation of the results obtained. This is essential to confirm the clinical diagnosis, predict prognosis, and determine appropriate genetic counseling. In addition, appropriate genotyping has profound implications for clinical management and treatment. Understanding the relationship between specific genetic variants and treatment outcomes is crucial for personalized medicine approaches in CAH.

## Figures and Tables

**Table 1:** SALSA MLPA Probemix P050-D1 CAH. *CYP21A2* probes detect the wild-type sequences of seven loci while *CYP21A1P* probes are specific for 4 variants mapping within promoter region, exon 3, exon 4, and exon 7, respectively. ■: this symbol refers to the CYP21A2 probe 22959-L32396 located within exon 4 of the gene. ▲: this symbol refers to the CYP21A2 probe 15221-L20261located within exon 3 of the gene. ►: this symbol refers to the CYP21A2 probe 17261-L21169 located within exon 7 of the gene. ▼: this symbol refers to the CYP21A2 probe 17270-L16990 located within exon 6 of the gene. ◄: this symbol refers to the CYP21A2 probe 17271-L16989 located within exon 6 of the gene. ◊: this symbol refers to the CYP21A2 probe 21552-L20299 located within intron 2 of the gene*. ○: this symbol refers to the CYP21A2 probe 21552-L32321 located within intron 2 of the gene*. ●: this symbol refers to the CYP21A2 probe 22964-L32402 located within promoter region of the gene. ♦: this symbol refers to the CYP21A1P probe 22961-L32398 located within exon 4 of the pseudogene. □: this symbol refers to the CYP21A1P probe 15221-L20262 located within exon 3 of the pseudogene. ◙: this symbol refers to the CYP21A1P probe 17261-L21170 located within exon 7 of the pseudogene. ⸙: this symbol refers to the CYP21A1P probe 22963-L32401 located within promoter region of the pseudogene. * The copy number detected by these two probes should be combined.

SALSA MLPA Probe	Exon Position
**Gene**	
(■) CYP21A2 probe 22959-L32396	Exon 4, p.(Ile173Asn) location
(▲) CYP21A2 probe 15221-L20261	Exon 3, p.(Gly111fs) location
(►) CYP21A2 probe 17261-L21169	Exon 7, p.(Leu308fs) location
(▼) CYP21A2 probe 17270-L16990	Exon 6, p.(Val238Glu) location
(◄) CYP21A2 probe 17271-L16989	Exon 6, p.(Met240Lys) location
(◊) CYP21A2 probe 21552-L20299 *	Intron 2, c. 293-13A allele
(○) CYP21A2 probe 21552-L32321 *	Intron 2, c. 293-13C allele
(●) CYP21A2 probe 22964-L32402	Promoter region, c.-113G>A location
**Pseudogene**	
(♦) CYP21A1P probe 22961-L32398	Exon 4, p.(Ile173Asn) location
(□) CYP21A1P probe 15221-L20262	Exon 3, p.(Gly111fs) location
(◙) CYP21A1P probe 17261-L21170	Exon 7, p.(Leu308fs) location
(⸙) CYP21A1P probe 22963-L32401	Promoter region, c.-113G>A location

## Data Availability

The original contributions presented in the study are included in the article, further inquiries can be directed to the corresponding author.
